# A Review of the CMOS Buried Double Junction (BDJ) Photodetector and its Applications

**DOI:** 10.3390/s8106566

**Published:** 2008-10-23

**Authors:** Sylvain Feruglio, Guo-Neng Lu, Patrick Garda, Gabriel Vasilescu

**Affiliations:** 1 University P. & M. Curie – Paris 6, SYEL – BC 252, 4 Place Jussieu, 75252 Paris Cedex 05, France E-mail: patrick.garda@upmc.fr.; 2 Institut des Nanotechnologies de Lyon (INL), CNRS UMR5270, University Claude Bernard Lyon 1, 43 Boulevard du 11 Novembre 1918, 69622 Villeurbanne Cedex, France E-mail: guo-neng.lu@univ-lyon1.fr; 3 City University London, DEEIE, UNESCO Chair, Northampton Square, London EC1V OHB, UK; E-mail: gabiv@neuf.fr

**Keywords:** Buried Double Junction photodetector, CMOS Image Sensor

## Abstract

A CMOS Buried Double Junction PN (BDJ) photodetector consists of two vertically-stacked photodiodes. It can be operated as a photodiode with improved performance and wavelength-sensitive response. This paper presents a review of this device and its applications. The CMOS implementation and operating principle are firstly described. This includes the description of several key aspects directly related to the device performances, such as surface reflection, photon absorption and electron-hole pair generation, photocurrent and dark current generation, etc. SPICE modelling of the detector is then presented. Next, design and process considerations are proposed in order to improve the BDJ performance. Finally, several BDJ-detector-based image sensors provide a survey of their applications.

## 1. Introduction

During the past two decades, CMOS technology has been increasingly exploited to develop integrated optical sensors in the Ultra-Violet (UV), visible and Near-Infrared (NIR) domains. One prominent example is the CMOS Image Sensors (CIS), which have emerged as alternate solid-state imaging devices to the mature Charge-Coupled Devices (CCD). Typically, CMOS sensors provide advantages such as low power, low cost and high system integration [1, 2]. In this context, a CMOS Buried Double Junction (BDJ) detector was proposed [3-20]. It has interesting features compared to the classical PN photodiode.

In this paper, we present a review of this optical detector and its main applications in CMOS optical sensor systems. The paper is organized in six sections. Following the introduction, Section II describes the BDJ detector structure and its operation principle. Section III presents the SPICE modelling of the device. In Section IV, design and process considerations are proposed in order to suggest ways to improve its performance. In Section V, several implementations and applications of the BDJ detector are given. Section VI concludes this paper.

## 2. The BDJ Photodetector Device

After a brief description of the BDJ structure, the principle of operation is presented by recalling several key concepts.

### 2.1. Structure and operation

**Figure 1.**
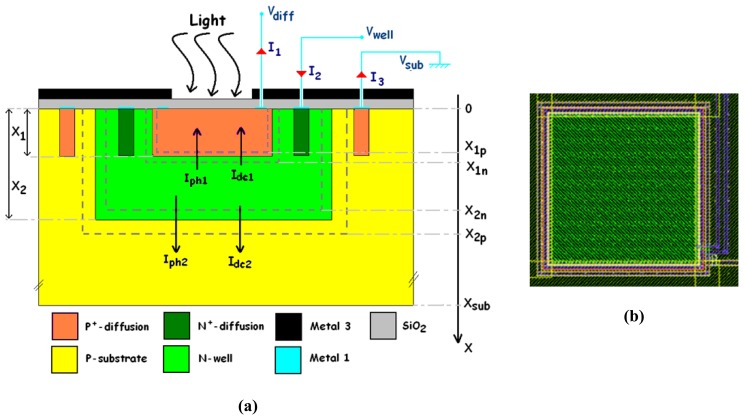
**(a)** Simplified cross-section of the generic BDJ PD and **(b)** layout of a square BDJ.

The device consists of two stacked buried photodiodes. Its implementation in a standard n-well CMOS process is illustrated in Figure 1a. It has a vertically stacked shallow P^+^-diffusion/N-well junction (J_1_) and a deep P-substrate/N-well junction (J_2_). The P-substrate is grounded, and two outputs correspond to the P^+^-diffusion and N-well contacts, respectively. A top metal layer is employed as an optical mask to define the active surface of the detector and to prevent radiation penetration in its peripheral area. The two buried PN junctions (J_1_ and J_2_) are reverse-biased, supplying two currents I_1_ and I_2_ to the outputs. Observe that the output current I_1_ flows through the shallow junction, and that the other output current I_2_ is the sum of the currents passing through both junctions.

To reverse-bias both junctions, the bias condition is V_well_ > V_diff_ > V_sub_ = 0 V, where V_diff_, V_well_ and V_sub_ are the potentials respectively applied to the P^+^-diffusion, the N-well and the P-substrate (see Figure 1.a).

Under illumination, the current of each junction has two components: a photo-generated component and a dark component. Thus, both output currents can be expressed as:

(1-a) $$I_{1}=I_{ph1}+I_{dc1}$$

(1-b) $$I_{2}=I_{ph1}+I_{dc1}+I_{ph2}+I_{dc2}=I_{1}+I_{3}$$

**Figure 2.**
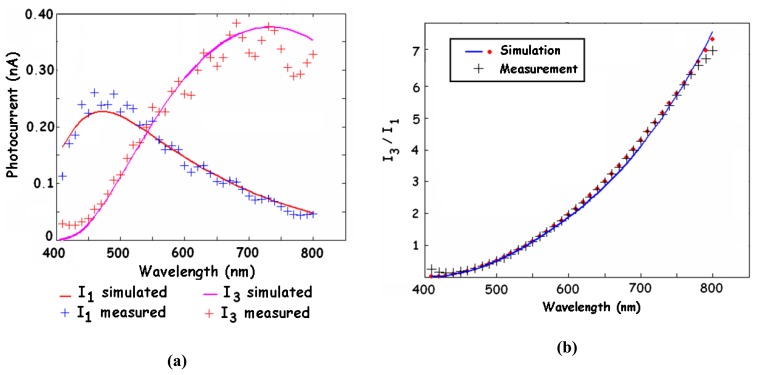
Spectral characteristics of a CMOS BDJ detector. **(a)** Spectral responses of the shallow and deep junctions; **(b)** photocurrent ratio I_ph2_/I_ph1_ versus wavelength.

The device operation is based on the strong wavelength dependence of the silicon absorption coefficient *α(λ)*[3-16, 22, 23, 26, 29-38]. Blue light is absorbed near the silicon surface while red light has a much deeper penetration. Consequently, the shallow junction has a sensitive response to blue light because it collects only photo-generated carriers near the silicon surface. On the other hand, the deep junction is sensitive to red and NIR light thanks to the carriers generated in the deeper region. The spectral responses of both junctions are shown in Figure 2a. Besides, measurements of both output currents allow estimation of the photocurrent ratio I_3_/I_1_. This ratio is monotonically increasing with the wavelength, as shown in Figure 2b. Therefore, the BDJ detector can be employed either as a colour- or as a wavelength-sensitive device [3-16, 20].

### 2.2. Surface reflection

Consider first the bulk silicon surface. Some part of the incident light is reflected. The transmitted part of a photon flux with identical energy can be expressed as:

(2) $$\Phi_{t}\left(\lambda \right)=\left(1-R\left(\lambda \right)\right)\Phi \left(\lambda \right)$$

where R(λ) is the reflection coefficient and Φ(λ) the incident photon flow. R(λ) is given by R = |r|^2^ where r is the reflection rate calculated from complex Fresnel coefficients [23]. It depends on the air and Si refraction coefficients.

**Figure 3.**
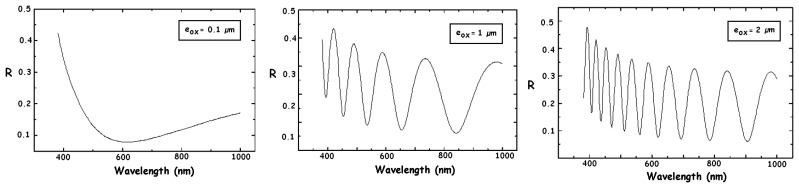
Simulation results of the reflection coefficient R versus wavelength for various SiO_2_ thickness e_ox_.

However, a CMOS detector has at least one SiO_2_ layer on its surface. The reflection index of this surface passivation layer differs from the air and silicon indices. This causes reflections at both the air/SiO_2_ and SiO_2_/Si interfaces. Since it is a thin layer, there are interference effects between these reflections, which result in oscillating variations of the effective surface reflection coefficient versus wavelength. Figure 3 shows such variations depending on the SiO_2_ thickness [3, 10, 27, 28].

The same effects can induce fluctuations on the spectral responses of the detector, as can be observed on the plots of Figure 2a. In the case of CMOS imagers, these reflections may lead to a loss of contrast and sharpness of the obtained images (reflections or “ghosts” on the image). From Equation (2), it can be seen that the reflected light is definitively lost by the detected signal. This leads to a decrease of the quantum efficiency. Solutions for minimizing the reflection effects include anti-reflect coating and a proper choice of the surface layer's thickness.

### 2.3. Light absorption in silicon and generation rate of electron-hole pairs

When a photon flux with energy hν ≥ E_G_ penetrates inside the silicon bulk, a photoelectric conversion process occurs: ideally, the absorption of one photon creates an electron-hole pair. The generation rate of the electron-hole pairs, at a certain depth from the silicon surface, has the following expression:

(3) $$g\left(x,\;\lambda \right)=\Phi_{t}\left(\lambda \right)\alpha \left(\lambda \right)\exp\left(-\alpha \left(\lambda \right)x\right)$$

**Figure 4.**
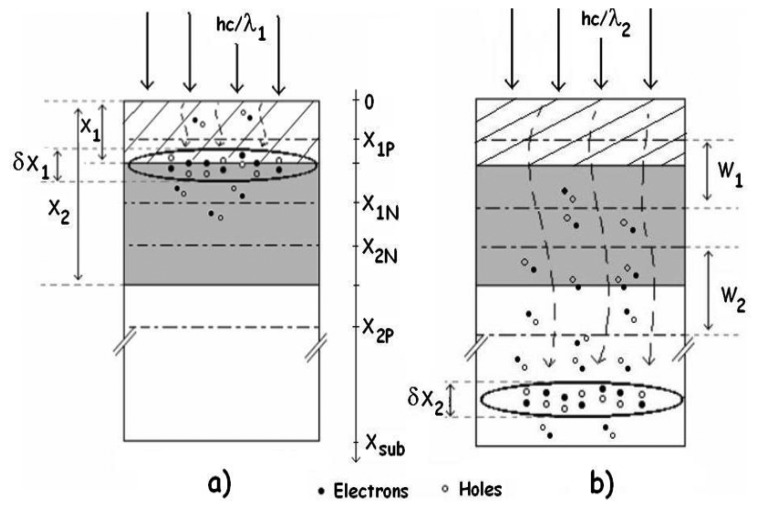
Photo-generated carriers inside the BDJ detector in the case of **(a)** blue light (λ∼450 nm) and **(b)** red light (λ ∼680 nm).

**Figure 5.**
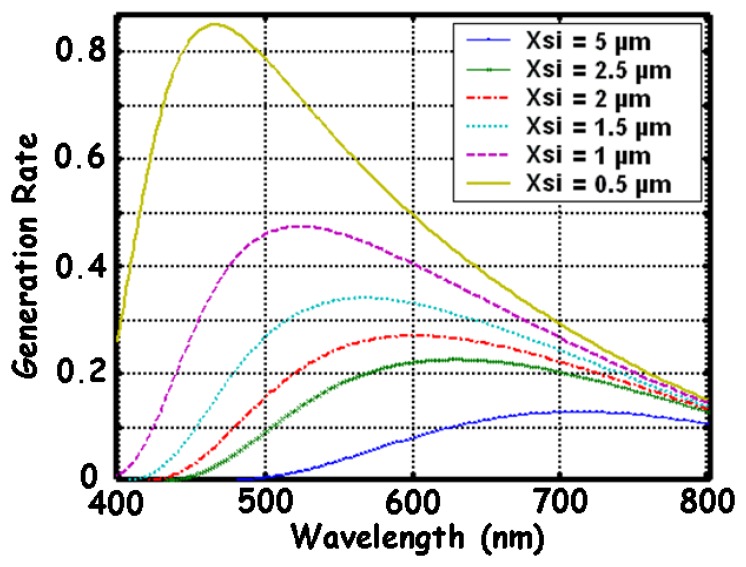
Simulation of the normalized generation rate of the electron-hole pairs versus wavelength, for several layers of intrinsic Si.

Since it includes the absorption coefficient α, it is dependent of the wavelength. For a given wavelength, the maximum generation rate is proportional to the absorption depth, i.e. × = α^-1^. This result can mathematically be obtained from Equation (3). It indicates a region depth where most photo-generated carriers are found. For blue light, this depth is about 0.2 μm, and for red light, it is about 5 μm. The region depth of the photo-generated carriers inside a BDJ detector is given as a function of the wavelength in Figure 4. Figure 5 shows the calculated generation rate as a function of the wavelength, for several depths in the silicon.

**Figure 6.**
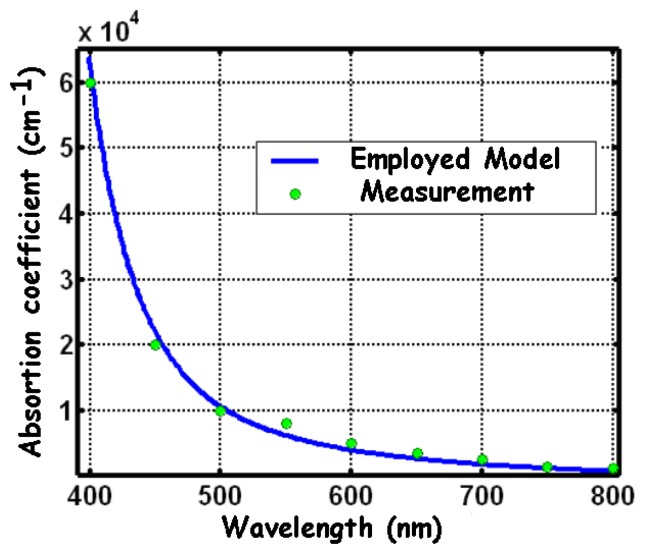
Absorption coefficient of the visible incident light versus wavelength, in silicon, at 298 K.

The absorption coefficient in Equation (3) is obviously a key parameter. It strongly depends on the wavelength and on the temperature. There are several analytical or empirical models of the absorption coefficient [29-38], among which the Geist model [29] seems to provide a better agreement with the experimental results at 298 K (see Figure 6). According to this model, the absorption coefficient, expressed in cm^-1^, can be calculated as:

(4-a) $$\alpha =\frac{\sum_{i=1}^{2}\sum_{\begin{array}{l} j=-1\\ j\neq 0 \end{array}}^{1}\left\{C_{ji}{\left[L\left(E-E_{k}+jdE_{i}\right)\right]}^{2}\right\}+C_{3}{\left[2L\left(E-E_{3}\right)\right]}^{N+\text{dNE}}}{E}$$

(4-b) $$\text{with}\;L\left(E\prime \right)=\frac{E\prime +\left|E\prime \right|}{2eV}=\left\{ \begin{array}{c} E\prime /1eV\;\text{for}\;E\prime >0\\ 0\;\text{for}\;E\prime \leq 0 \end{array}\right.$$

The expression (4-a) includes eleven fitted parameters [29], based on [30, 31]: C_-11_=5030.02 cm^-1^; C_11_ = 483.916 cm^-1^; C_-12_=1634.30 cm^-1^; C_21_ = 79.4079 cm^-1^; C_3_=1046.08 cm^-1^; E_k_ = 1.09969 eV; E_3_ = 1.40985 eV; dE_1_ = 0.0583148 eV; dE_2_ = 0.0220161 eV; N = 0.394122; dN = 1.23084 eV^-1^.

This equation is an approximation to the theoretical analysis of McLean [30]. The double sum in Equation (4) describes the indirect transition from the parabolic region near the maximum of the valence band to the parabolic region near the minimum of the conduction band. The sum is composed of four terms to describe the emission (j = -1) and the absorption (j = +1) of TO phonons (with energy dE_1_) and TA phonons (with energy dE_2_). Finally, the last term in this equation is an attempt to describe the smooth increase beyond the quadratic dependence, as described by McLean. This increase is expected from the increasing density of states in the conduction band due to the deviation of the bands from parabolicity at the higher energies.

To take into account the temperature effect, one may to compute the absorption coefficient by combining this model and experimental data at different temperatures with interpolation techniques [35, 37]. For instance, one may to determine α, by interpolation at any temperature between T_1_ and T_2_, is to use the following relationship:

(5) $$\alpha \left(T\right)=\alpha \left(T_{2}\right){\left[\frac{\alpha \left(T_{1}\right)}{\alpha \left(T_{2}\right)}\right]}^{\frac{T_{2}-T}{T_{2}-T_{1}}}$$

Doping also has an effect on the absorption coefficient. Heavily doped semiconductor may lead to a significant increase of α, due to the band gap narrowing on one hand, and to the absorption contribution of the free carriers on the other hand. However, such an effect may not be noticeable for a doping level lower than 10^18^ atomes/cm^3^[38].

### 2.4. Photocurrents

Under reverse-bias conditions, each junction collects photo-generated charges inside and around its depletion region. The resulting photocurrent across the junction thus has two components: a drift current and a diffusion current [3, 4, 7, 10, 11, 20, 22-24, 26, 40, 43]. The drift component corresponds to charges collected in the depletion layer, and the diffusion current results from charges collected from both quasi-neutral adjacent regions.

The collection process inside the depletion layer of the junction is fast and effective: due to the internal electric field, photo-generated holes and electrons are rapidly separated and flow in opposite directions. Almost all these charges contribute to the photocurrent without being affected by electronhole recombination, which is a much slower process. The drift current can thus be calculated by integrating the generation rate over the depletion layer [3, 4, 7, 10, 11, 19, 20, 22, 24, 26, 40, 43]:

(6) $$I_{dr\theta }\approx qA_{j}\Phi_{t}\exp\left(-\alpha W_{\theta }\right)$$

where q is the elementary charge; A_j_ is the active surface area; θ = 1 or 2 depending on the considered current and W_1_ and W_2_ denote the depletion-layer widths.

The diffusion of minority carriers in the quasi-neutral adjacent region toward the depleted region border is a relatively slow process. Due to the recombination, only photo-generated carriers within a diffusion length from the depletion border can be collected and they contribute to the photocurrent. To determine the diffusion currents inside the BDJ detector, the concentration of excess minority carriers should first be calculated by solving the continuity equation with appropriate boundary conditions [3, 4, 7, 10, 11, 19, 20, 24, 26, 40, 43]. Diffusion component can then be determined for any kind of layer by:

(7-a) $$I_{\text{phD}}=qA_{j}D_{p}{\frac{\partial p_{n}}{\partial x}|}_{x}$$

(7-b) $$\text{or}\;I_{\text{phD}}=-qA_{j}D_{n}{\frac{\partial n_{p}}{\partial x}|}_{x}$$

where D_p_ and D_n_ represent the diffusion coefficient for holes and electrons respectively; p_n_ and n_p_ represent the concentration of excess minority carriers in the observed quasi-neutral region.

Let the minority current through the P^+^ region be denoted I_phD11_, while the minority current through the P-substrate will be I_phD22_. Inside the N-well, two opposite hole diffusion components (I_phD12_ and I_phD21_) can be put into evidence. Finally, the following general expression was obtained:

(8) $$I_{\text{phD}\theta \theta \prime }=qA_{j}\Phi_{t}S_{\theta \theta \prime }$$

where θ, θ′ = 1 or 2, depending on the considered component; S_θθ′_ denotes the diffusion quantum efficiency of the region of interest, which is a function of the geometrical parameters (x_1P_, W_1_, etc.), the surface recombination velocity S_r1_ (estimated to 10^7^ cm/s, for simulation purposes), the radiation wavelength, the doping profile, etc.

After evaluation of all components, both photocurrents across the shallow and the deep junction can be respectively deduced from the following relationships:

(9-a) $$I_{ph1}=I_{\text{phD}11}+I_{\text{phD}12}+I_{dr1}$$

(9-b) $$I_{ph2}=I_{\text{phD}21}+I_{\text{phD}22}+I_{dr2}$$

**Figure 7.**
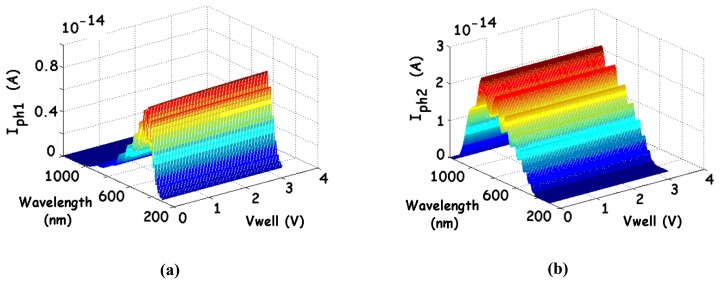
Matlab computed photocurrents of the BDJ detector versus the wavelength and the reverse bias, considering the reflection coefficient R in SiO_2_: **(a)** Photocurrent of the shallow junction J_1_ and **(b)** Photocurrent of the deeper junction J_2_.

Figure 7 shows an example of the I_ph1_ and I_ph2_ computed photocurrents, for a given incident light intensity (1 μW.cm^-2^). Their variations versus the wavelength represent the spectral responses. The parameters correspond to a 20 μm × 20 μm BDJ detector designed in a 0.35-μm CMOS AMS technology. Since bias voltages are also included in these computations (to determine the depletion-layer depths), the effects of such parameters on the global response can be evaluated. As it appears from Figure 7, the bias voltages have not a strong influence on the photocurrents.

In the same way, the temperature dependence of I_ph1_ and I_ph2_ can be estimated, provided that the computations include accurate models of the temperature-sensitive parameters such as the absorption coefficient [see Equation (5)], the diffusion coefficient, the carriers' mobility, the intrinsic carrier concentration, etc. In [3], both simulations and measurements show that the peak of I_ph1_ shifts towards long wavelengths as the temperature increases (a 30 nm variation is estimated between the peaks at 223 K and 338 K) and the spectral response of J_1_ increases with temperature. For the photocurrent I_ph2_, the same results were observed.

**Figure 8.**
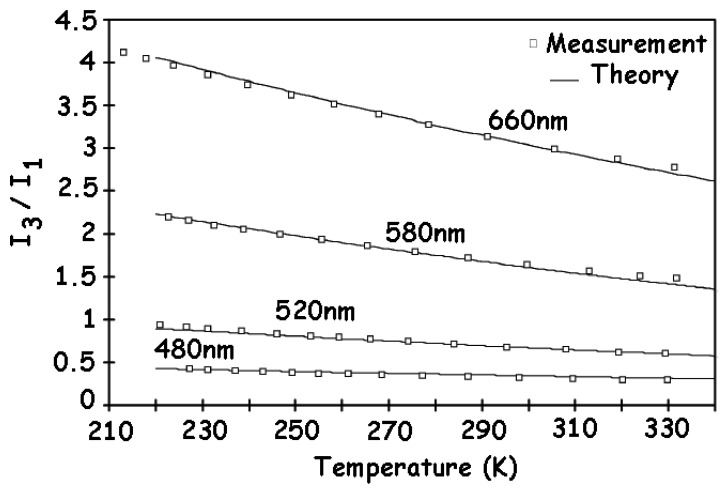
Photocurrent ratio I_3_/I_1_ versus temperature [3].

When it operates as a wavelength-sensitive detector, the photocurrent ratio is also an important characteristic of the BDJ sensor. Its temperature-dependence predicted by theoretical computations is confirmed by experimental measurements [3]. Figure 8 depicts an excellent agreement of the results. The photocurrent ratio shows a steady decrease as the temperature increases.

To compensate temperature effects, it is required to implement some on-chip temperature measurements. One simple solution suggests the use of an N-well resistance as a thermistor [3].

### 2.5. Response time

In many applications, the response time of the detector to the incident light signal is a benchmark that must be carefully considered and analyzed.

As already mentioned, the photocurrent of a junction has two components: the drift current and the diffusion current. The former has a faster response than the latter, as the charges collecting process differ. Whereas the diffusion current contributes substantially to the overall current (typically in long-wavelength detection), the response time of the detector is primarily set by the diffusion process [3, 21, 25]. Thus, Sedjil et al. [3] studied the impulse response (ideally a Dirac δ(t)) of diffusion currents in quasi-neutral regions, in order to deduce the response to a light pulse (a Heavyside step U(t)). During this very brief moment of illumination, it is assumed that the photo-carriers do neither have time to recombine, nor time to diffuse. Therefore, the density of minority carriers established in each region can lead to the expression of the corresponding diffusion photocurrent component. Since the pulse response is the product of the convolution between the impulse response and a Heavyside step, it can be shown that the time constant of each quasi-neutral region is [3]:

▪ (10-a) $$\text{For the}\;{\text{P}}^{+}‐\text{diffusion}:\frac{1}{\tau_{\text{diff}}}=\frac{1}{\tau_{n1}}+{\left(\frac{\pi }{2x_{1p}}\right)}^{2}D_{n1}$$
  which yields τ_diff_ comprised between 0.13 ns and 0.15 ns, with a CMOS 0.8μm technology;
▪ (10-b) $$\text{For the}\;\text{N}‐\text{well},\;\text{we have}:\;\frac{1}{\tau_{\text{Nwell}}}=\frac{1}{\tau_{p}}+{\left(\frac{\pi }{2\left(x_{2n}-x_{1n}\right)}\right)}^{2}D_{p}$$
  τ_Nwell_ is in the range [0.14 ns; 0.16 ns];
▪ (10-c) $$\text{For the P substrate},\;\text{we note that}:\frac{1}{\tau_{\text{sub}}}=\frac{1}{\tau_{n2}}+{\left(\frac{\pi }{2\left(x_{\text{sub}}-x_{2p}\right)}\right)}^{2}D_{n2}$$

This corresponds to a time constant τ_sub_ between 1 ns and 0.8 μs.

From these equations, we note that the time constants of the quasi-neutral regions depend not only on the lifetimes of the minority carriers (τ_n1_, τ_n2_ and τ_p_) and the minority carrier diffusion constants (D_n1_, D_n2_ and D_p_), but also on the depths of the layer (x_1p_, x_1n_, x_2n_, x_2p_ and x_sub_). If the thickness of these layers is considered negligible, the lifetime has little influence on the time constant. But for large thicknesses, this time must be taken into account.

An experimental study of the response time of this detector structure has been reported in [25]. With CMOS 1-μm technology and a light wavelength of 783 nm, both rise time and fall time of about 8 ns were observed for the deep junction J_2_. The surface junction J_1_ has a shorter response time (about 0.1 ns) because of high doping. These results are typically better than those of a CMOS conventional photodiode, mainly because the BDJ detector has two buried depletion regions instead of one, thus effectively reducing the slow diffusion contribution. In addition to this improvement of the response time, the BDJ detector also has an improved quantum efficiency resulting from its more effective charges collection in silicon.

### 2.6. Dark currents

Without illumination, the leakage current flowing through a reverse-biased PN junction is called dark current (denoted I_dc_). This current depends on the junction characteristics and is superimposed to the photocurrent across the junction. It places a limit on the integration duration for measuring the light intensity and it generates a dark noise [19, 20, 46-47]. The value of dark current imposes a physical limit on the two critical benchmarks of any photodetector (PD): the optical detection range and the noise floor.

The particular value of the dark current varies from sample to sample and depends on the temperature, the fabrication quality, the doping level, the bias voltage, the physical design (layout), etc. It is assumed to be mainly due to the thermally generated carriers, but it can be seriously increased due to defects of the semiconductor lattice, dislocations, and unwanted contamination by impurities during fabrication. Furthermore, for image sensors, the dark current may exhibit large variations from pixel to pixel. This dark current non-uniformity increases the Fixed Pattern Noise (FPN) and the Dark Signal Non-Uniformity (DSNU) [19, 20, 48, 49].

**Figure 9.**
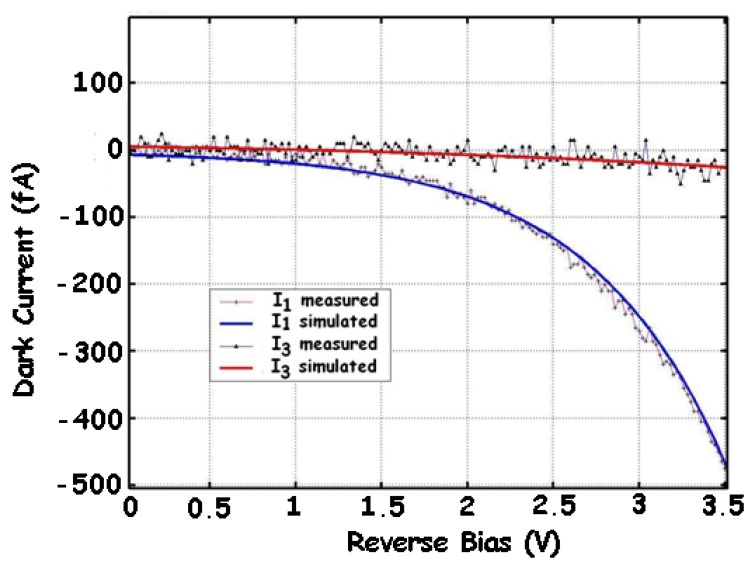
Measured and simulated I–V plots of J_1_ and J_2_ junctions in dark, for a 50 × 50 μm^2^ BDJ at 300 K [17-20].

**Figure 10.**
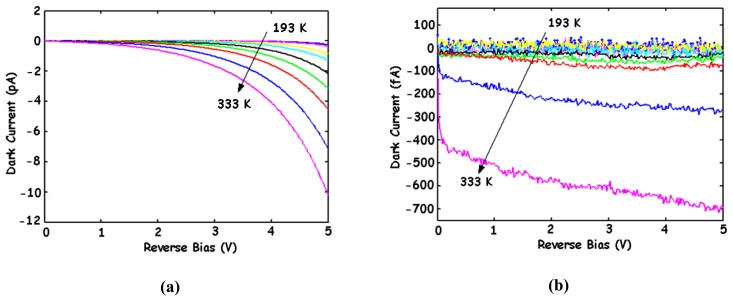
Measured dark current of **(a)** the shallow junction J_1_ and **(b)** the deeper junction J_2_, at various temperatures, for a 50 × 50-μm^2^ BDJ [51].

Many physical processes may be involved in the generation of I_dc_. The most important are the diffusion process, the thermal generation of carriers (Shockley-Read-Hall model), the Band-to-Band Tunnelling (BBT), the Trap-Assisted Tunnelling (TAT) and the impact ionization [17-20, 22, 23, 26, 39, 50-52]. The contributions of these processes depend on the technological and geometrical parameters of the device, the temperature and the bias conditions. The diffusion component may become significant at high temperatures, while the contribution of the thermal generation is related to the depletion-layer width. These two contributions may be identified by their weak dependence on the bias voltage, and their strong dependence on the temperature. In the cases of high-doping and high-bias-voltage conditions, the tunnelling and ionization effects may dominate, which leads to a rapid increase of I_dc_ with the bias voltage. Figure 9 shows the simulated and measured results of both dark currents of a BDJ detector in a 0.35-μm CMOS process. For J_1_, the dark current exhibits a sensitive increase with the reverse bias voltage, indicating that the diffusion and thermal generation for low voltages (< 1.5 V) and BBT for high voltages (> 1.5 V) are respectively the dominant processes. For J_2_, the diffusion and thermal generation are the two main contributions, even under a reverse bias up to 3.5 V. The dark current measurements at different temperatures (Figure 10) further confirm this statement [39, 51, 52]. The observed BBT effect on J_1_ is predictable, because this junction is built with a heavily doped P^+^-diffusion layer. Finally, both dark currents of the BDJ detector can be expressed as [17-20]:

(11-a) $$I_{dc1}=A_{j1}\left(J_{g1}+J_{d1}+J_{t1}\right)$$

(11-b) $$I_{dc2}=A_{j2}\left(J_{g2}+J_{d2}\right)$$

where A_j1_ and A_j2_ are the areas of junctions J_1_ and J_2;_ J_g1_ and J_g2_ denote the thermal generation current densities; J_d1_ and J_d2_ correspond to the diffusion (dark) current densities of J_1_ and J_2;_ J_t1_ is the BBT current density of J_1_.

To avoid the sharp increase of I_dc1_ due to the BBT effect, a low reverse bias voltage for J_1_ is chosen.

## 3. BDJ SPICE model

The successful design of integrated optical sensor based systems, such as APS for CMOS digital cameras, position sensitive detectors or flame detection, depends on the complete and accurate description of the behaviour of the optical sensor. As simulations are compulsory, an accurate model of the employed photodetector must be found.

A general SPICE model of the BDJ photodetector was proposed for large-, small-signal operations and noise analysis [10, 17-20, 53-55]. An overview is presented in this section, together with its implementation in CAD tools.

### 3.1. Large-signal, small-signal and noise models

#### 3.1.1. Large-signal model

**Figure 11.**
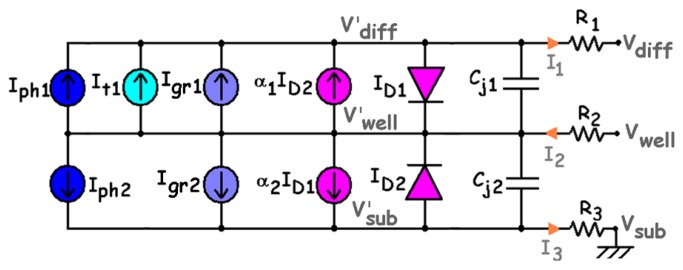
Large-signal equivalent circuit of the BDJ PD.

Large-signal operation corresponds to sensor operation either in steady-state or transient state with an intensive incident radiation. The large-signal equivalent circuit is presented in Figure 11, where R_1_, R_2_, R_3_ are parasitic (non-linear) resistances; C_j1_, C_j2_ denote the junction capacitances; I_ph1_, I_ph2_ correspond to the photo-generated currents. The components of I_dc1_ and I_dc2_ taken into account are: the thermal G-R components I_gr1_, I_gr2_; the BBT current denoted I_t1_. I_D1_, I_D2_ stand for the diffusion currents and α_1_I_D2_, α_2_I_D1_ are the current-controlled current sources performing the mutual coupling between the junctions, like in any Ebers-Moll model of bipolar transistors [17-20, 40, 57].

The parasitic resistances correspond to the ohmic contacts and the bulk resistances of the quasi-neutral zones. They reduce the sensor efficiency and slightly modify the junction intrinsic bias (such as ${\text{V}}_{\text{diff}}^{\prime }={\text{V}}_{\text{diff}}+{\text{R}}_{1}{\text{I}}_{1}$, ${\text{V}}_{\text{well}}^{\prime }={\text{V}}_{\text{well}}-{\text{R}}_{2}{\text{I}}_{2}$ and ${\text{V}}_{\text{sub}}^{\prime }={\text{V}}_{\text{sub}}+{\text{R}}_{3}{\text{I}}_{3}$). They can be estimated from the sheet and contact resistances (according to the foundry datasheet), from geometrical considerations [57] or by measurement. Thus, for a 20 × 20-μm^2^ BDJ sensor active area (in the configuration of Figure 1.b), with V_diff_ = V_sub_ = 0 V and V_well_ between 0 V and 3.3 V, the worst-case computed parasitic series resistances are around 3 Ω for the P^+^-diffusion region, 35 Ω for the N-well layer and 7 kΩ for the P-substrate.

Concerning the junction capacitances (C_j1_ and C_j2_, in Figure 11), as the transition capacitances are negligible under the reverse bias condition, only the non-linear diffusion capacitances are considered. According to [17-20, 26, 58], the junction capacitances can be computed with the following equations:

(12) $$C_{j\theta }=\frac{A_{j\theta }C_{jS0\theta }}{{\left(1-\frac{V_{\theta }}{V_{bi\theta }}\right)}^{M_{jS\theta }}}+\frac{P_{j\theta }C_{\text{jSW}0\theta }}{{\left(1-\frac{V_{\theta }}{V_{bi\theta }}\right)}^{M_{\text{jSW}\theta }}}=\frac{C_{j0\theta }}{{\left(1-\frac{V_{\theta }}{V_{\text{biEFF}\theta }}\right)}^{M_{j\theta }}}\approx \frac{\varepsilon_{0}\varepsilon_{Si}A_{j\theta }}{W_{\theta }}$$

where the newly introduced parameters are: P_jθ_, the perimeter of the considered junction (θ = 1 for J_1_ and 2 for J_2_); C_jS0θ_, the zero-bias junction area capacitance; C_jSW0θ_, the zero-bias sidewall capacitance; C_j0θ_, the zero-bias effective capacitance; V_biθ_, the built-in potential; V_biEFFθ_, the effective built-in potential; M_jSθ_, M_jSWθ_ and M_jθ_, dimensionless technological parameters depending on the doping profiles of both PN junctions (with values around 1/2 and 1/3 for the abrupt and the linear junctions respectively); *ε*_0_*ε_Si_*, the silicon permittivity, ${\text{V}}_{1}=V_{\text{well}}^{\prime }-V_{\text{diff}}^{\prime }$ and ${\text{V}}_{2}=V_{\text{well}}^{\prime }-V_{\text{sub}}^{\prime }$.

Note that in the last forms of Equation (12), the area and sidewall contributions are not separated and the capacitance value is estimated with respect to the effective width of the depletion layer [19-20].

#### 3.1.2. Small-signal model

Small-signal operation implies low or variable intensity radiation impinging on the sensor surface. It is usually employed to describe the alternate (dynamic) behaviour of the device due to a perturbation of infinitesimal amplitude, which does not shift the operating point.

As the junction dark and photo-generated currents depend on the optical sensitivities and on the applied bias, the adopted approach consists in taking the exact differential of each DC current equation [17-20]. Obviously, a function depending on the intrinsic junction voltages ( $V_{\text{well}}^{\prime }-V_{\text{diff}}^{\prime }$ and $V_{\text{well}}^{\prime }-V_{\text{sub}}^{\prime }$) and on the incident optical flow Φ_t_ results.

(13) $$\{\begin{array}{c} i_{1}=g_{11}\left(v_{\text{well}}^{\prime }-v_{\text{diff}}^{\prime }\right)+g_{12}\left(v_{\text{well}}^{\prime }-v_{\text{sub}}^{\prime }\right)+s_{1}\Phi_{t}\\ i_{2}=i_{1}+i_{3}\\ i_{3}=g_{21}\left(v_{\text{well}}^{\prime }-v_{\text{diff}}^{\prime }\right)+g_{22}\left(v_{\text{well}}^{\prime }-v_{\text{sub}}^{\prime }\right)+s_{2}\Phi_{t} \end{array}$$

**Figure 12.**
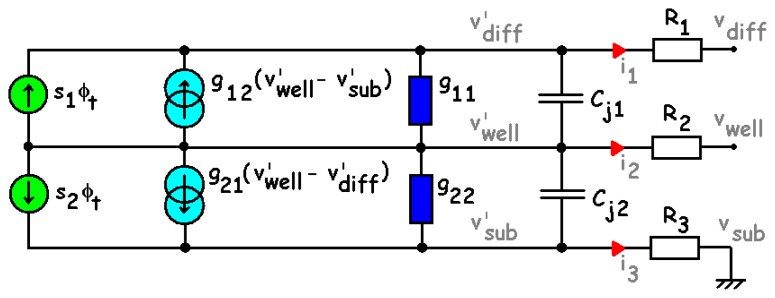
Small-signal equivalent circuit of the BDJ sensor.

**Figure 13.**
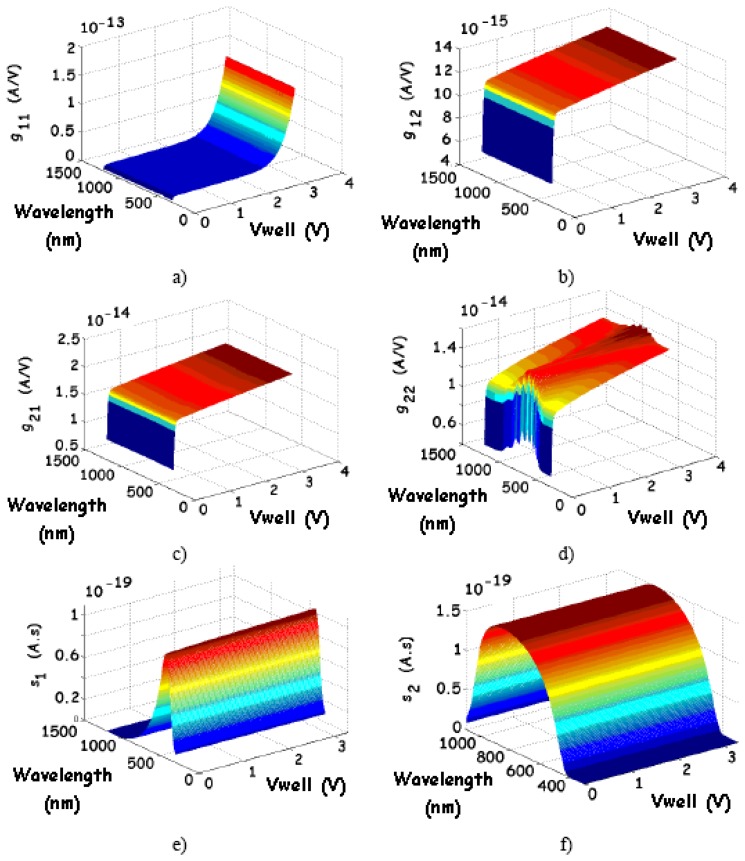
Simulated AC parameters of the BDJ PD (under the same conditions than in Figure 7).

The small-signal equivalent circuit is presented in Figure 12, where, according to Equations (13), the following notation has been adopted: g_11_ and g_22_ are the dynamic conductances; g_12_ and g_21_ are the transconductances and s_1_ and s_2_ are the optical sensitivities (or spectral responses). For a 20 × 20-μm^2^ sensor in the same conditions as in Figure 7, we find the following maximum values: g_11_ ≈ 1.4·10^-13^ S; g_22_ ≈ 1.5·10^-14^ S; g_12_ ≈ 1.4·10^-14^ A/V and g_21_ ≈ 2.0·10^-14^ A/V. As shown in Figures 13.a to 13.d, with an incident red light, the conductance is mainly influenced by the dark and photo-generated G-R current components. Concerning J_1_, as long as λ is high, the influence of the incident light is not visible and, therefore, only the contribution of the dark current is clearly put in evidence. In contrast, for J_2_, since V_well_ > 0, g_22_ is monotonically increasing with V_well_ and it varies with the radiation wavelength λ consistent with the J_2_ spectral response. Moreover, for a uniform light as adopted to get the plots of Figure 7, maximum values of 0.5·10^-19^ A.s.cm^2^ and 1.49·10^-19^ A.s.cm^2^ have been respectively obtained for s_1_ and s_2_ (Figures 13.e and 13.f).

#### 3.1.3. Noise model

**Figure 14.**
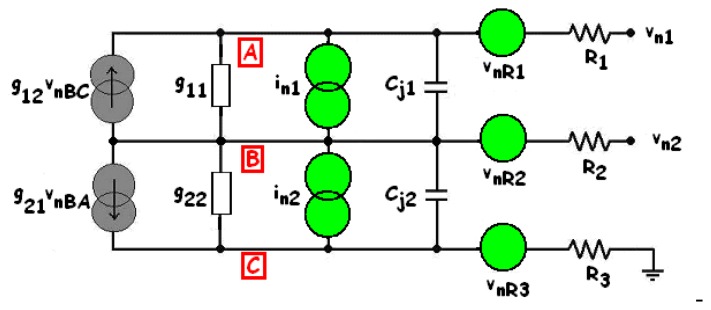
Equivalent noise circuit of the BDJ PD.

The intrinsic electrical noise arises inside the device. It superposes onto the useful signal and tends to obscure its informational content. Considering the various noise mechanisms, the noise equivalent circuit of the BDJ PD (Figure 14) was obtained. It includes three thermal noise voltage generators v_nR1_, v_nR2_ and v_nR3_ to account for the thermal fluctuations arising in the parasitic resistances. It also involves two equivalent current noise generators, denoted I_n1_ and I_n2_, placed at each junction and lumping the effects of the shot, G-R and 1/f noise components. The spectral densities are calculated using the general equations [13, 17-20, 46, 54-56, 59]:

(14) $$S_{\text{VnR}}\left(f\right)=\frac{\bar{v_{nR}^{2}}}{\Delta f}=4\text{kTR}$$

(15) $$S_{In}\left(f\right)=\frac{\bar{i_{n}^{2}}}{\Delta f}=2q\left|I\right|+4q\left|I\right|\frac{\tau_{T}}{\tau_{d}}\frac{1}{1+4\pi^{2}f^{2}\tau_{T}^{2}}+K\frac{{\left|I\right|}^{\sigma }}{f^{k}}$$

where I corresponds to the DC currents flowing through the considered junction; τ_T_ is the relaxation time of the traps; τ_d_ denotes the transit mean lifetime of the carriers; f is the frequency of the light radiation; K, σ and κ are device-dependent constants. Typically, we note that 0.5 < σ < 2, 0.8 < κ < 1.3 and K is a constant obtained by fitting experimental data.

### 3.2. Models implementation in CAD tools

The design of mixed signal systems on chip is nowadays a challenge, especially those including APS for imaging purposes. To simulate such systems, it is necessary to use optoelectronic models of the PDs together with a high level simulator. Conventional circuit simulators, such as SPICE, do not provide models for optoelectronic devices. In order to by-pass this limitation, circuit designers must adopt some tricks that reduce the accuracy. In particular, a simple PN photodiode is normally modelled as a current source, having only a constant capacitance in parallel. In the best case, we have a photocurrent source, modelled from a voltage-controlled current source (where “voltage” is interpreted as the incident power light), in parallel with a diode. However, even if the capacitance and the parasitic resistance of the PD are successfully modelled, the simulation of the photocurrent or dark current behaviours are not satisfactory [18-20, 61-67]. Thus, this simplified approach, although good enough for some applications, is often not suitable to simulate the circuits whose performance is critically dependent on the PD characteristics. To overcome this problem, a rigorous multi-domain approach is generally recommended.

As a first attempt, we have employed SPICE, where a macro-model of PD (using sub-circuits) was proposed to model photocurrents [10-12, 64-67]. After some practice, it appeared clearly that the employment of multi-domains languages, such as Verilog-A and VHDL-AMS, could be very useful. Actually, this approach is the most employed in order to simulate single PD and equally complex Optical System On-Chip (OSOC), having analog and digital parts, with relatively low simulation time [41-44, 68-72]. In this context, the recent availability of the SystemC-AMS language [73, 74] seems to offer the better perspectives for the simulation of complex systems.

Nevertheless, in order to improve the simulation accuracy, various optical sources must also be developed to improve the flexibility of the model implementation in various applications and to allow more realistic simulations. Generally, the optical spectrum is assumed to be white or monochromatic. However, other optical stimuli are possible (light star, incandescent lamps, etc.) and various optical power distributions are possible. For instance, a PD is usually coupled with a LED or a laser, which have a quasi-gaussian spectrum [12, 62, 70, 71].

## 4. Design and process considerations

This section presents different approaches and considerations to optimize device characteristics, such as noise and dark currents, and spectral responses. Some suggestions point optimal design and proper choice of a given CMOS technology, others require additional process steps. Here, we will mainly focus on the former.

### 4.1. Solutions to reduce dark currents

Firstly, some efforts are needed to minimize the effects of SiO_2_/Si interface states. They may generate surface dark current and related shot noise as well as 1/f noise. They may also degrade the blue light response of the detector through surface recombination process. Experimentally, a good rule is to choose P^+^-diffusion/N-well/P-substrate instead of N^+^-diffusion/P-well/N-substrate structure, since a high concentration of holes near the silicon surface neutralizes the interface states' effects.

**Figure 15.**
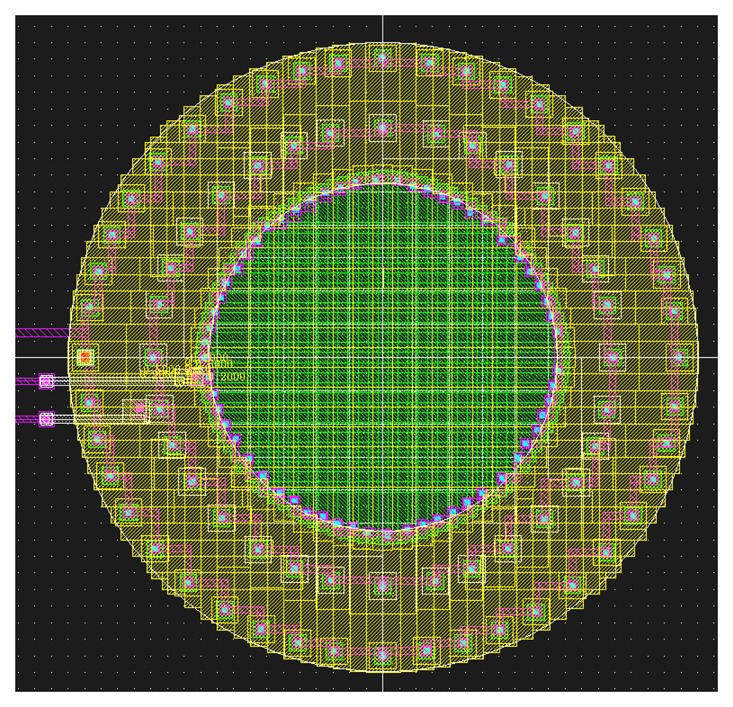
Layout of a circular BDJ PD, where low resistance access, low sidewall component and low crosstalk have been optimized at the expense of the Fill Factor (FF).

Another rule is to minimize as much as possible the perimeter-to-area ratio. Especially for small-size devices, dark currents are typically dominated by contributions from surface depletion regions [39]. Thus, to minimize side-wall peripheral contribution, instead of adopting BDJs of square-shape, circular or hexagonal shapes are more convenient [75]. Figure 15 shows an example of circular BDJ layout. Circular layout also minimizes BBT effect [76] and junctions capacitances.

Other considerations include effects of some manufacturing steps inducing stress, etching damages etc. For instance, the use of Deuterium annealing instead of the conventional gas annealing process shows good perspectives [77]. Hydrogen annealing was also proved to reduce leakage by passivating the defects [78] In contrast, the use of Shallow Trench Isolation (STI) [79], thin gate oxide, and salicide cause high dark current. To reduce the non-silicided dark current, double-diffused source/drain implantation is used in Active Pixel Sensor (APS) [80].

Aiming at dark current reduction, low bias voltages can be chosen [17-20, 39, 81] and cooling implementation may be considered such as the use of Peltier devices. However, to compensate the temperature effects, some on-chip temperature measurements have to be implemented, by means of thermistors [3].

### 4.2. Solutions to improve quantum efficiency and Signal-to-Noise Ratio

As it has already been pointed out in Section 2, the spectral response of a buried junction depends on its depth. Consequently, according to the wavelength of interest, the choice of the technology should take into account this aspect. Alternatively, Wolffenbuttel et al. propose to control the spectral sensitivity by modulating the depletion areas (W_1_ and W_2_) of both stacked PN junctions [15]. The width of the depletion layers increases with the reverse bias (V_well_-V_diff_ and V_well_-V_sub_) applied. Hence, their reverse voltages can tune the collecting volumes of both PN junctions. As can be seen from Figure 7, a significant shift of response can be obtained in the region between 360 and 500 nm provided that the width of the depletion layers can vary in the order of 1 μm or more. However, this depletion-width modulation effect can be sensitive only in low doping cases (typically, less than 10^-15^ cm^-3^). However, in the most usual submicron CMOS processes, layers with low doping levels are not available.

The most effective optimization is at the process level. Specific implant to control the doping profiles of J_1_ and J_2_ may increase quantum efficiency at visible wavelengths and also lower capacitance. Deposition of anti-reflect coating and microlens may improve quantum efficiency. Also, as suggested by Simpson et al. [82], the responsitivity of PDs can also be controlled by using the poly-Si and glass coatings as thin-film optical filters and also by controlling the density of the traps at the interface Si/SiO_2_. However, these techniques are expensive, requiring specific process optimization and integration.

It is recognized that the use of a shallow junction and a high doping results in a low quantum efficiency. A possible solution, for high visible wavelength, is to use a BiCMOS Buried Triple Junction (BTJ) PD [3-6, 11, 45, 83, 84], where only the middle and the deep junctions are used or, more simply, a P^-^/N-well/P-sub structure. Moreover, it is admitted that the multi-layer structure increases the quantum efficiency of PDs compared to the simple conventional PN junctions, even for poor quality Si [85-90]. Hence, to increase the quantum efficiency, various SiON materials are being tried out to increase light transmission. Non-silicided deep junctions (such as N-well/P-Sub or N-diff/P-Sub) are generally used [91]. In addition, as it was previously suggested, in connection with our test structures (Figure 1), a metal employed as an optical mask is highly recommended.

Like any photo-sensor, the BDJ detector exhibits several noise sources: shot noise, G-R noise and 1/f noise. It was proved [13, 19, 20] that the Power Spectral Density (PSD) of both the shot and G-R noises increases with the junction current, while, for the 1/f noise, it is a quadratic function of the junction DC current. When the signal level increases, shot (or G-R) noise dominates, but the SNR will still be increased because the photocurrent increases at a faster rate than the noise. At low illumination levels, the 1/f noise may have a significant contribution and all solutions, to reduce it, should be considered. It is well-known that for a zero-voltage bias, the 1/f noise contribution vanishes. However, with zero bias, the PD is rather in the photovoltaic mode, which is not appropriate. Kleinpenning et al. [59] stated that the 1/f noise is proportional to the carriers' lifetime, which, in turn, depends on the doping ( $∼\sqrt{N}$, for an abrupt junction). Thus, the corner frequency, between the shot noise and the flicker noise, decreases with the increasing lifetime. It follows that a lower doping level may help to reduce the 1/f noise. Nevertheless, this possibility is contrary to the actual tendency of continuous downscaling in standard CMOS technologies, where PN junctions at very small depth are made of higher doping layers.

By considering the output signal as the ratio of photocurrents delivered by the BDJ detector, one way to define the SNR is [13]:

(16) $${\text{SNR}}_{I_{3}/I_{1}}^{-1}\propto \frac{\Delta \left(I_{ph2}/I_{ph1}\right)}{I_{ph2}/I_{ph1}}\approx \frac{I_{ph1}^{2}}{\bar{i_{n1}^{2}}}+\frac{I_{ph2}^{2}}{\bar{i_{n2}^{2}}}$$

Under the assumption that the fluctuations of both photocurrents are uncorrelated, expression (16) shows that this SNR couldn't be higher than those of photocurrents. However, when the noise of the incident light source is dominant, a simultaneous sampling of both output currents is recommended to cancel the common fluctuation part. Consequently, the SNR defined by Equation (16) will be improved.

As far as the deeper junction J_2_ is concerned, a highly doped epitaxial layer could also be associated with the P-substrate. Thus, the parasitic bulk resistance R_3_ in Figure 11 will be lowered and the substrate noise will be mitigated. In this way, Wang *et al*. [91] have implemented their BDJ detector on a high resistivity floating-zone silicon substrate by using ion implantation and this physical structure exhibits low leakage currents.

**Figure 16.**
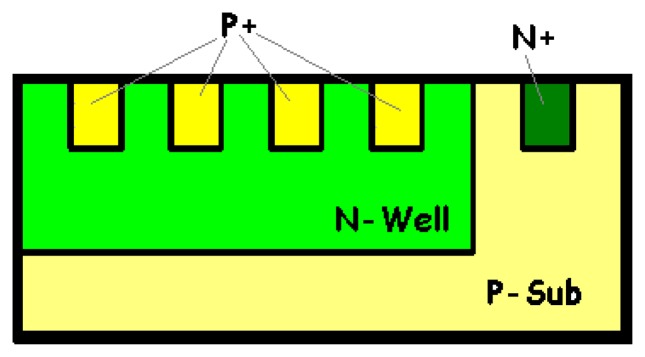
Simplified cross-section of the UV/Blue improved BDJ PD [93-95].

**Figure 17.**
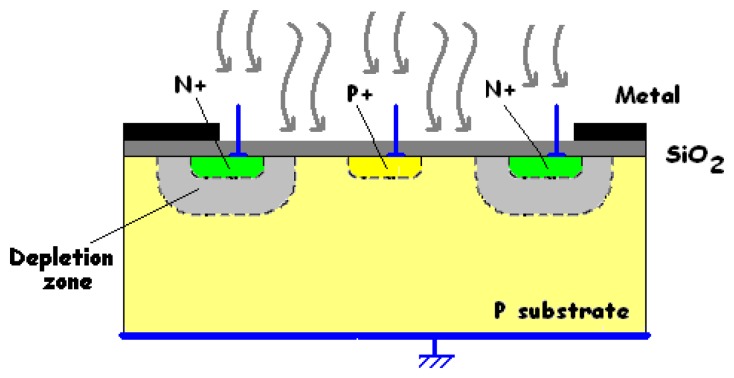
Simplified cross-section of the large-band light sensor proposed by A. Pinna [85].

**Figure 18.**
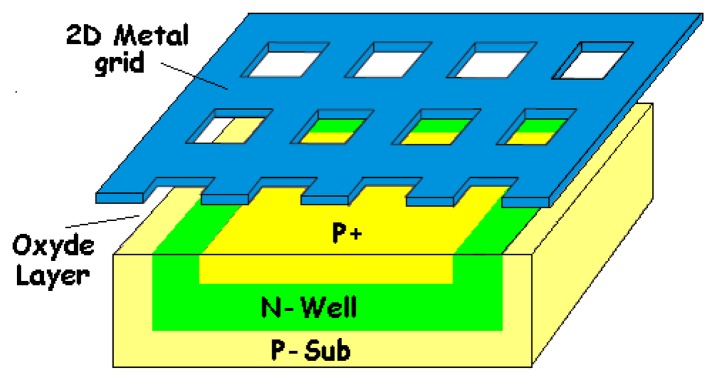
Associating the BDJ PD with a two-dimensional (2-D) metal grid [96].

In order to get better spectral characteristics, some techniques were proposed. To improve the short-wavelength responsivity, some authors [93-95] presented a BDJ detector with a strip-shaped anode geometry (for the shallow junction) to improve UV to green wavelength response. They used interdigitated P^+^-diffusion fingers to increase the depletion region area near the silicon surface (shown in Figure 16). The idea is similar to that of a lateral photodiode [43], to improve the collecting efficiency of photo-generated carriers near silicon surface. Another valuable idea to obtain a wide spectrum responsitivity was suggested by Pinna [85]. He proposed a structure consisting of two phototransistors, one vertical, the other lateral (see Figure 17). In reference [96], Yang *et al*. present a method for improving the spectral sensitivity of PDs manufactured in commercial 0.18-μm silicon CMOS technology, without the addition of post-processed colour filters. Similarly to microwaves techniques, combining the BDJ detector and metallic grids on its surface (Figure 18) enhances the sensitivity by an additional control of the spectral sensitivity and by shifting the region of high sensitivity to longer wavelengths. Note that Findlater *et al*. [97-99] designed a CMOS colour image sensor based on two BDJ detector pixels with deposited colour filters. In this way, four different spectral responses were obtained, which enabled tri-chromatic colour detection.

In practice, various detector sizes are required by specific applications. High-resolution imaging devices call for small-size implementation. In standard CMOS technology, the P^+^-diffusion layer is surrounded by N-well. Due to peripheral spacing rules imposed by the technology, the Fill Factor (FF) will deteriorate rapidly when the pixel pitch is limited to a few micrometers. However, it is possible (with advanced technologies) to bury thin oppositely-doped layers at different depths for collecting photocarriers [101]. The deep buried layer will not envelop the shallow one and in this way avoids peripheral spacing limitation and thus allows further reduction of pixel size. On the other hand, when very large size detectors are required, another limitation is set by the condition that the bulk substrate potential must not vary along the structure. In practice, for the bias of high-resistivity layers (such as N-well and P-substrate), multi-point contacts between each 100 μm or so should be provided to maintain equipotentiality. As bias contacts for P-substrate and N-well cannot be made in the central area of the detector, a possible solution for a large-size detector is to design an array of parallel-connected pixels.

## 5. Examples of applications in integrated CMOS optical sensors

This section presents examples of BDJ-based integrated systems especially for imaging applications. Such developments primarily exploit the spectral characteristics of the BDJ detector and its double operations enabling intensity and wavelength detection.

**Figure 19.**
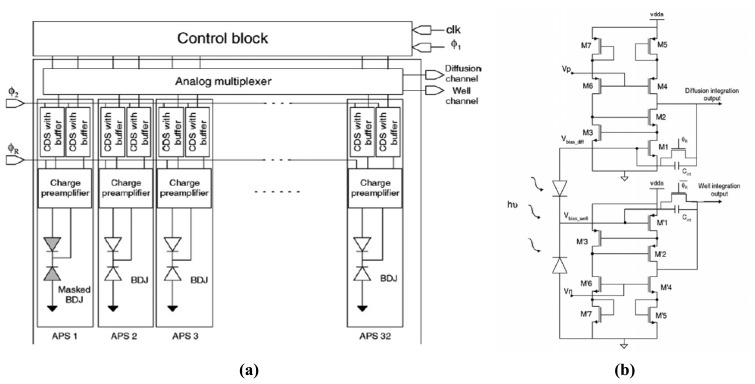
**(a)** Block diagram of the CMOS APS linear array and **(b)** schematic of one pixel with its two-channel charge preamplifier [102].

One example, shown in Figure 19, is a CMOS APS linear array [102, 103], dedicated to be a self-wavelength calibration micro-spectrophotometer. Each pixel integrates a BDJ detector both for light intensity measurement (like a photodiode) and for wavelength estimation of the received monochromatic component. The 32-active pixels linear array is designed to meet high-sensitivity and low-resolution requirements. The in-pixel electronic circuit includes a two-channel charge preamplifier and correlated-double-sampling (CDS) stages, as shown in Figure 19.

The BDJ detector has also gained interest in many 2D imaging systems [3, 4, 20, 53-55, 78, 97-99, 104, 105]. In a project developing colour image sensors, Findlater *et al*. [98-100] employed two BDJ-based pixels instead of 3 or 4 conventional PD-based pixels for tri-chromatic colour detection. The idea of this example is to exploit the integrated colour filtering properties of the BDJ detector. We can see from Figure 2a that the shallow junction has a selective response for blue light, while the deep junction exhibits a high sensitive response for red and NIR light. Combining such spectral responses with colour filter deposition on top of the two pixels, one may measure the colour by applying a tri­chromatic method. This approach, compared to 3 or 4-pixel colour imaging, presents several improvements in spatial frequency, resolution and colour accuracy. In [106-108], De Graaf *et al*. proposed a microsystem for illumination source identification.

**Figure 20.**
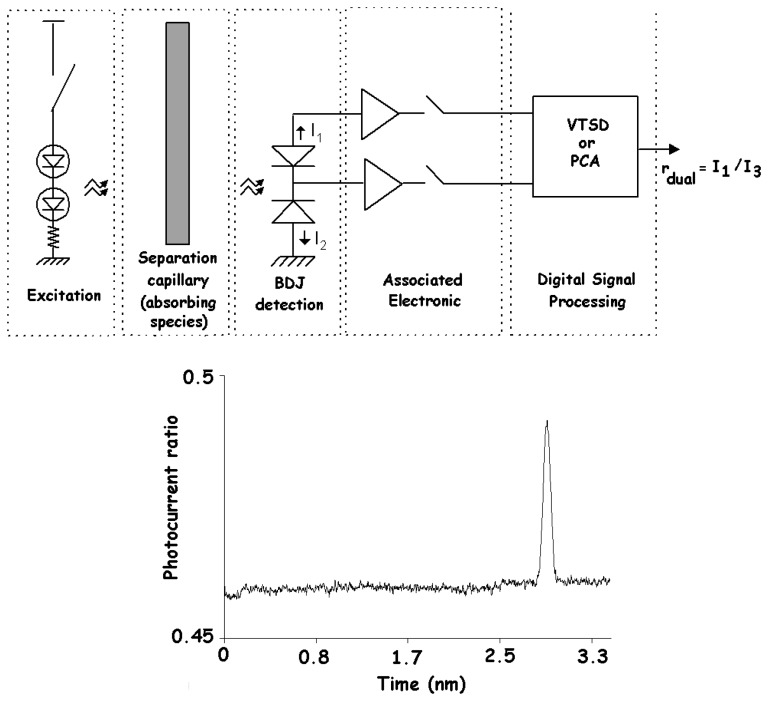
Capillary absorptiometric measurement with dual-wavelength method [110].

One important feature of the traditional BDJ detector lies in the photocurrent ratio evaluation by measuring its two outputs. Considering the spectral sensitivities plotted in Figures 2a and 2b and remembering that the surface reflection (see Section 2.2) may induce fluctuations on the spectral responses but has no effect on the photocurrent ratio, this may be a key issue in some particular applications. More generally, the fluctuations of the incident light intensity will not affect the photocurrent ratio, provided that both detector outputs are synchronously red [9, 13]. On the other hand, the photocurrent ratio can be exploited both to estimate the wavelength in the case of a monochromatic light source and to detect the spectral variations for the non-monochromatic excitation. This led to the developments of specific optical methods for detecting spectral changes without any dependence on the intensity variations. Figure 20 shows a dual-wavelength method recommended for a quantitative analysis via absorptiometric measurements [110-112].

The operation of the BDJ detector is particularly attractive to implement the optical detection for biochemical analysis. Taking fluorescence detection as an example, its photodiode mode can serve to quantify the species by measuring the fluorescence intensity, while its wavelength-detection mode allows spectral discrimination in the multi-label cases or detected signal estimation [112].

**Figure 21.**
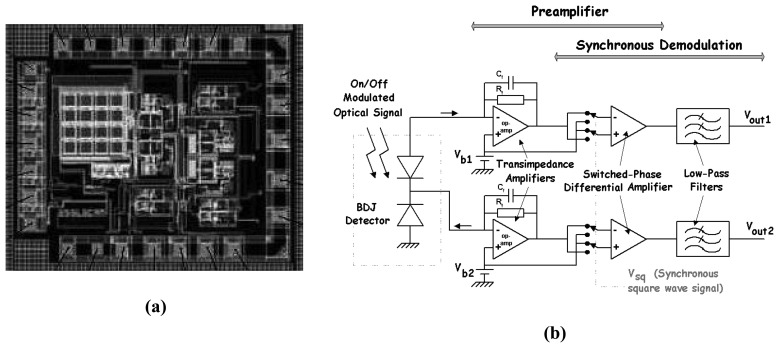
**(a)** Chip integrating an array of 4 × 4 BDJ pixels: **(b)** on-chip charge / transimpedance preamplifier and synchronous detection [112, 113].

To implement the micro-channel and micro-capillary detection, Figure 21 presents a chip integrating a 4 × 4-array of BDJ pixels. It enables either individual pixel reading or parallel connections of all the pixels operated as a single BDJ detector. For such applications, a large-area detector for the micro-channel adaptation of the optical-fiber-coupling is typically employed and the custom design of associated electronics is suggested to improve the SNR. The custom design allows the on-chip implementation of some noise reduction techniques, such as charge/transimpedance preamplifier, a synchronous detection (see Figure 21b) [110, 112] or high-gain differential approaches [116]. The chip of Figure 21a was used for capillary fluorescence detection. The evaluated limit of detection was below 10^-10^ M in fluorescein isothiocyanate (FITC) concentration for a small volume of 5 nL [114].

**Figure 22.**
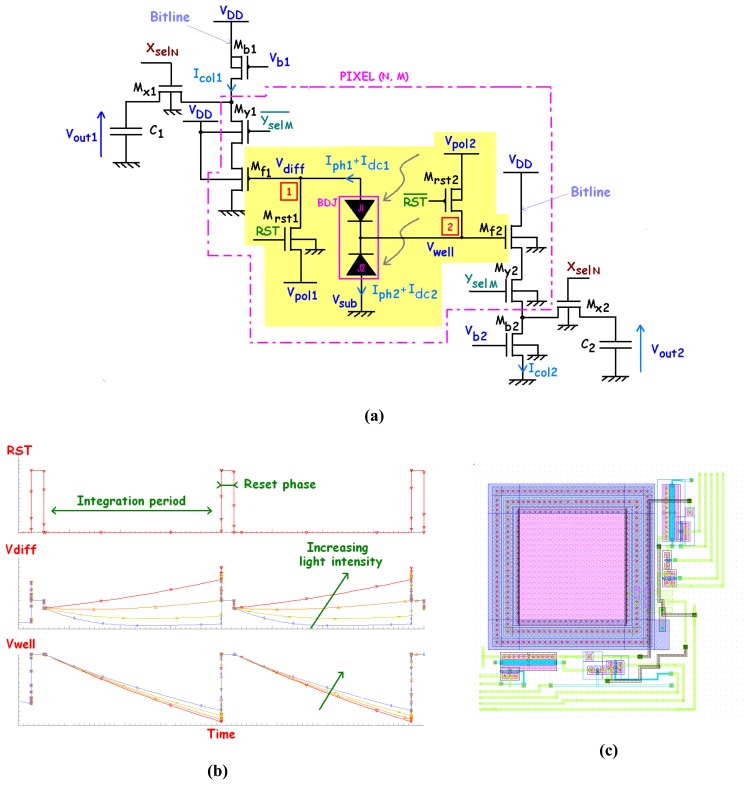
**(a)** CMOS BDJ APS architecture, **(b)** its corresponding timing diagram obtained from simulation and **(c)** its layout.

For 2D imaging applications requiring a good spatial resolution, the APS solution is an obvious choice. Many studies on in-pixel BDJ-associated electronics were reported [20, 53-55, 97-99]. Figure 22 shows an active pixel with simultaneous reset, integration and read operations for both channels. This architecture makes use of 6-transistors pixel. Thanks to its synchronous operations for both channels, the obtained photocurrent ratio will not be affected by the temporal fluctuations of the incident light intensity. This architecture can also be improved. For instance, a better FF may be obtained by sharing the same readout circuit between two neighbour pixels. Two shutters (i.e., MOSFETs) can be introduced between the BDJ outputs and the source follower transistors to isolate photo-generated signals (before or after reset transistors [117]). Moreover, according to [104-105], BDJ could be employed in 3D image reconstruction, where the first junction J_1_ provides the visible response and the second J_2_ the IR response.

## 6. Conclusions

In this paper, the state of the art of the BDJ optical sensor has been presented. It puts in evidence that this sensor, realized in standard CMOS technology, presents a real interest for low-cost and low-power applications. Since the BDJ provides two responses in a single pixel, this detector presents a better performance than any conventional photodiode. It is obvious that the BDJ photodetector can find a wide range of applications, such as micro-instrumentation (biomedical, chemistry, drug detection, etc.), communication systems, image sensors and more.
